# IDENTIFYING CARE ACTIVITIES THAT WERE SUPPORTED BY AMAZON ECHO FOR CARE PARTNERS AFTER ONSET OF COGNITIVE IMPAIRMENT

**DOI:** 10.1093/geroni/igz038.1194

**Published:** 2019-11-08

**Authors:** Galina Madjaroff

**Affiliations:** University of Maryland Baltimore County, Baltimore, Maryland, United States

## Abstract

There are several important challenges when addressing the needs of older adults with cognitive impairment and their care partners including the potential for diminishing emotional well-being and loss of autonomy, which could potentially lead to a lower overall quality of life for both care partners (CPs). The motivation of this study was to identify the care activities that were supported by home based technology for care partners after the onset of cognitive impairment. This work was done through gathering multiple sources of qualitative and quantitative data, including mobile application dialogue history logs, pre and post interviews, user feedback groups and home visits. The technology deployed in the home of the care partners was a Voice User Interface Intelligent Agent, specifically the Amazon Echo with its intelligent agent “Alexa.” This technology was selected because it was not built from a traditional care model, yet embodies functions that could be used for all potential forms of care, including those that achieve a higher level of quality of life goals for care partners. From this study, we can further our understanding of how to deploy and design technology that shifts the perspective from “cure to care” with a focus on the older person and their lived experience, monitoring wellness, and not just addressing illness. Results and findings indicated that daily care activities of dyads that are seemingly fundamental are actually complex care activities that emerge from using the technology that support the care partners on multiple levels in satisfying multiple needs.

